# Deciphering the Anticancer Arsenal of *Piper longum*: Network Pharmacology and Molecular Docking Unveil Phytochemical Targets Against Lung Cancer

**DOI:** 10.7150/ijms.98393

**Published:** 2024-07-22

**Authors:** Venkatramanan Varadharajan, Ashwath Kumar Balu, Atul Shiju, Pandiyan Muthuramalingam, Hyunsuk Shin, Baskar Venkidasamy, Naiyf S. Alharbi, Shine Kadaikunnan, Muthu Thiruvengadam

**Affiliations:** 1 Department of Biotechnology, PSG College of Technology, Peelamedu, Coimbatore, India.; 2 Division of Horticultural Science, College of Agriculture and Life Sciences, Gyeongsang National University, Jinju 52725, Korea.; 3 Department of Oral and Maxillofacial Surgery, Saveetha Dental College and Hospitals, Saveetha Institute of Medical and Technical Sciences (SIMATS), Saveetha University, Chennai 600077, India.; 4 Department of Botany and Microbiology, College of Science, King Saud University, P. O. Box 2455, Riyadh 11451, Saudi Arabia.; 5 Department of Crop Science, College of Sanghuh Life Science, Konkuk University, Seoul, Republic of Korea.

**Keywords:** Computational screening, Drug discovery, Medicinal plant compounds, Bioactivity, Oncogenes

## Abstract

**Introduction:** Lung cancer, characterized by uncontrolled cellular proliferation within the lung tissues, is the predominant cause of cancer-related fatalities worldwide. The traditional medicinal herb *Piper longum* has emerged as a significant contender in oncological research because of its documented anticancer attributes, suggesting its potential for novel therapeutic development.

**Methods:** This study adopted network pharmacology and omics methodology to elucidate the anti-lung cancer potential of *P. longum* by identifying its bioactive constituents and their corresponding molecular targets.

**Results:** Through a comprehensive literature review and the Integrated Medicinal Plant Phytochemistry and Therapeutics database (IMPPAT), we identified 33 bioactive molecules from *P. longum*. Subsequent analyses employing tools such as SwissTargetPrediction, SuperPred, and DIGEP-Pred facilitated the isolation of 676 potential targets, among which 72 intersected with 666 lung cancer-associated genetic markers identified through databases including the Therapeutic Target Database (TTD), Online Mendelian Inheritance in Man (OMIM), and GeneCards. Further validation through protein-protein interaction (PPI) networks, gene ontology, pathway analyses, boxplots, and overall survival metrics underscored the therapeutic potential of compounds such as 7-epi-eudesm-4(15)-ene-1β, demethoxypiplartine, methyl 3,4,5-trimethoxycinnamate, 6-alpha-diol, and aristolodione. Notably, our findings reaffirm the relevance of lung cancer genes, such as CTNNB1, STAT3, HIF1A, HSP90AA1, and ERBB2, integral to various cellular processes and pivotal in cancer genesis and advancement. Molecular docking assessments revealed pronounced affinity between 6-alpha-diol and HIF1A, underscoring their potential as therapeutic agents for lung cancer.

**Conclusion:** This study not only highlights the bioactive compounds of *P. longum* but also reinforces the molecular underpinnings of its anticancer mechanism, paving the way for future lung cancer therapeutics.

## Introduction

Lung cancer, also known as lung carcinoma, is characterized by uncontrolled proliferation of cells within lung tissues 1,2. The prevalence of lung cancer is particularly high in North America, Europe, and East Asia, with China accounting for more than one-third of all lung cancer cases worldwide. In contrast, South Asia and Africa exhibit significantly lower rates of lung cancer 3. This disease is one of the primary causes of cancer-related mortality worldwide, including the United States. Lung cancer is the foremost habitually analyzed frame of cancer and the driving cause of cancer-related passings 4. Lung cancer is significantly influenced by age, with older individuals being more vulnerable to it. The incidence of lung cancer in patients aged < 45 years is minimal, and most cases occur in individuals aged ≥65 years. On an average, lung cancer is typically diagnosed at approximately 70 years of 70 5.

Smoking causes 85-90% of lung cancer cases and is primarily responsible for this development. The chemicals released from tobacco smoke damage the lung tissue, increasing the risk of lung cancer. Exposure to second-hand smoke increases the risk of lung cancer. It is crucial to remember that both smokers and nonsmokers can develop lung cancer, and that some cases can occur in people without any established risk factors. Often referred to as "non-smoker's lung cancer," this particular type of lung cancer seems to strike women and those of Asian ancestry more commonly 5. Various treatment modalities are available for lung cancer, including surgery, radiation therapy, chemotherapy, targeted therapy, immunotherapy, and palliative care 6. Early stage lung cancer is typically treated through surgical intervention, whereas chemotherapy and radiation therapy are employed in the later stages of the disease 7. In cases where specific genetic mutations are present, targeted therapy and immunotherapy can be utilized as viable treatment options.

Network pharmacology is a scientific discipline that investigates the intricate interactions between drugs and biological systems. The primary objective is to identify drug targets and elucidate the systemic mechanisms of drugs 8. To achieve this, computational and data-driven methods, such as machine learning, network analysis, and bioinformatics, have been employed to analyze and interpret molecular and pharmacological information, thereby comprehending the complex interactions between drugs and biological targets. Network pharmacology plays a crucial role in drug discovery and development and in the study of disease mechanisms. Using this approach, novel drug targets can be identified, aiding the development of new drugs that are more effective and specific. It also enables the evaluation of drug efficacy based on interactions with biological systems, assessment of drug toxicity, and repurposing of existing drugs. These applications facilitate the drug development process, leading to a reduction in associated costs and mitigation of side effects. Phytochemical constituents of medicinal plants have recently become the focus of increasing interest in the field of network pharmacology.

*Piper longum*, a member of the Piperaceae family comprising approximately 3,600 species, is commonly referred to as pippali or Indian long pepper. This flowering plant is indigenous to the Indian subcontinent and is cultivated in tropical regions such as South America, Africa, and Madagascar. *P. longum*, which has long, slender spikes with tiny berry-like fruits, is picked and dried for use as a spice and in traditional medicine 8. When a plant is fully grown, it becomes a shrub with expanded nodes, numerous creeping stems and substantial woody roots. *P. longum* fruits hold significant value in Ayurvedic medicine and are renowned for their therapeutic effects on respiratory, digestive, and reproductive health. They also possess anti-inflammatory, antioxidant, and analgesic properties. Analgesic, antidepressant, anti-amoebic, anti-obesity, radioprotective, cardioprotective, hepatoprotective, immunomodulatory, anti-microbial, anti-platelet, anti-hyperlipidemic, and antifungal properties are just a few of the beneficial characteristics that make up the pharmacological profile 9. *P. longum* is an important ingredient in many Ayurvedic preparations used to treat various ailments, such as leprosy, tuberculosis, cough, shortness of breath, heart and spleen disorders, persistent fever, gout, and rheumatic pain 10.

Network pharmacology analysis was used in this study to predict effective inhibitors of lung cancer-related targets using phytochemical ingredients extracted from the roots and fruits of *P. longum*. The analysis included several critical steps such as collecting and organizing information about the chemical compounds found in *P. longum*, building a network using analytical tools, identifying key nodes through network analysis, validating results, and interpreting findings to identify potential drug targets and therapeutic applications. This procedure is time consuming and requires expertise in pharmacology and computational biology. Network pharmacology analysis is a potent approach for exploring the intricate interactions between natural compounds and biological systems. Our study has the potential to identify new therapeutic strategies for lung cancer.

## Materials and Methods

### Screening for bioactive compounds in* P. longum*

Bioactive compounds present in the Root and Fruit of *P. longum* through literature mining 11, coupled with the utilization of the IMPPAT (Indian Medicinal Plants, Phytochemistry and Therapeutics) database (https://cb.imsc.res.in/imppat/) with “*Piper longum*'' as keywords. All compounds and their canonical sequences were retrieved from PubChem (https://pubchem.ncbi.nlm.nih.gov/).

### Identification of potential bioactive compounds of* P. longum*

The bioactive compounds identified in the earlier step underwent ADME analysis using the SwissADME server (http://www.swissadme.ch/) 12. Swiss ADME was used to evaluate the pharmacokinetics, drug-likeness, and medicinal chemistry compatibility of the small compounds. ADME, an abbreviation in pharmacokinetics, encompasses the processes of absorption, distribution, metabolism, and excretion, playing a pivotal role in drug discovery. Oral bioavailability (OB) is a critical pharmacokinetic measure that indicates a drug's capacity to enter systemic circulation following oral delivery. Drug-likeness (DL) refers to the similarity between a chemical and existing medication. OB and DL were used as the primary parameters for screening the active constituents. To adhere to the ADME criteria, specific restrictions were imposed, such as an overall OB ≥ 30% 13 and the exclusion of violations pertaining to Lipinski's rule of 5, to filter the phytochemical components.

### Identification of potential targets of* P.longum*

Target interactions of bioactive compounds were investigated using SwissTargetPrediction (http://www.swisstargetprediction.ch/) 14, SuperPred (https://prediction.charite.de/) 15, and DIGEP-Pred (http://www.way2drug.com/ge/) 16. Target selection was conducted using the SwissTargetPrediction tool with a p-value greater than 0.4. For SuperPred, targets with a p-value exceeding 80 were chosen, whereas for DIGEP-Pred, targets with a p-value higher than 0.5 were considered 17.

### Screening for targets in lung cancer

Various databases, including the Therapeutic Target Database (https://db.idrblab.net/ttd/), OMIM (https://www.omim.org/) (with a probability threshold > 0.4) 18, and GeneCards (https://www.genecards.org/) (with a relevance score exceeding 30) 19, have been used to identify the target proteins associated with lung cancer 20.

### Overlapping targets for compound targets and disease targets

Venny 2.1.0 (https://bioinfogp.cnb.csic.es/tools/venny/) was used to identify common targets between compound and disease targets. The intersection of the identified targets reveals important information about the relationships of the target with bioactive substances.

### Protein-protein interaction (PPI) network construction and analysis

Protein-protein interactions (PPIs) are of paramount importance in governing cellular functions and biological processes in various organisms. Understanding protein interactions can enhance our understanding of infection mechanisms and facilitate the development of effective drugs and treatment strategies. A protein-protein interaction (PPI) network was built using the STRING database (https://string-db.org/), focusing on specific genes with high-confidence interaction scores of ≥ 0.7 21. Subsequently, the network data obtained from the STRING database were extracted in a tab-separated value file format to build the protein-protein interaction (PPI) network diagram and perform enrichment analysis using Cytoscape v3.9.1 software 22.

### Compound, Target and Disease (C-T-D) network construction and analysis

A C-T-D (C-T-D) network was constructed using Cytoscape v3.9.1 software (https://cytoscape.org/). The nodes representing diseases, drugs, and core genes associated with the disease and compounds were extracted. Through node analysis, multiple targets were identified based on their degree of connectivity, reflecting their significance. Color and node-size scaling techniques were employed to depict the entire network based on the number of connections. The largest node represents the component with the highest number of connections. The CytoNCA plugin was used to conduct network topology analysis, and the component-disease target network map was imported into Cytoscape v3.9.1. The components were subsequently arranged in ascending order of importance based on their degree 23, 24.

### Gene Ontology (GO) enrichment and pathway analysis

ShinyGO 0.7 (http://bioinformatics.sdstate.edu/go/) 25 was used for pathway and process enrichment analysis. This program includes a standard set of enrichment analysis ontologies, such as Gene Ontology (GO) processes, Kyoto Encyclopedia of Genes and Genomes (KEGG) pathways, reactome gene sets, canonical pathways, and comprehensive resource of mammalian protein Complexes) complexes. GO biological process enrichment analysis and KEGG pathway analysis were performed on the target genes identified in the protein-protein interaction (PPI) network 26.

### Gene expression analysis

The gene expression profiling interactive analysis dataset (GEPIA 2, http://gepia.cancer-pku.cn/) 27 was used to analyze the expression of the top five hub genes (lung cancer genes). Lung cancer-associated gene expression levels in Lung Adenocarcinoma (LUAD) and Lung Squamous cell carcinoma (LUSC) compared to normal cells. According to Muthuramalingam *et al.*, the log2FC and p-values were set to 1 and 0.01, respectively, in the GEPIA 2 webserver 28.

### Overall survival and survival heat map analysis

Overall survival and survival map analysis of the top five lung cancer genes were validated against LUAD and LUSC in-built datasets using the GEPIA 2 server, which was used to predict the impact of patients' survival time 29. Survival analysis was performed using the Kaplan-Meier plotter in GEPIA2 with the preset default parameters.

### Molecular docking and visualization

The PDB identifier of the top five previously identified core targets was obtained through literature mining, and their corresponding PDB structures were downloaded from the Protein Data Bank (PDB) (https://www.rcsb.org/). The downloaded target structure files are shown in .pdb format were then accessed using Swiss-PDB Viewer (SPDBV) software. Prior to energy minimization, water molecules and other heteroatoms such as Ca, Cl, and Na were removed from the structures. Next, the top five bioactive compounds (ligands) from *P. longum*, represented by their canonical SMILES notation, were converted into their respective compounds .sdf file format using the OSIRIS DataWarrior (https://openmolecules.org/datawarrior/). Subsequently, the target molecules in the PDB format were loaded into PyRx software (https://pyrx.sourceforge.io/), and the software's auto dock setting was employed to convert them into macromolecules, which were then saved as .pdbqt files. The drug molecules were saved earlier .sdf file format, was inputted using the Open Babel module, energy-minimized, and converted to Autodock Ligand (.pdbqt). The VinaWizard window was opened and both macromolecules and ligands were selected for docking. Finally, the protein-ligand interactions resulting from the docking process were analyzed using the BIOVIA Discovery Studio Visualizer 2016 v16.1.0.15350 28.

## Results

### Active bioactive compounds in* P. longum*

**Figure 1:**
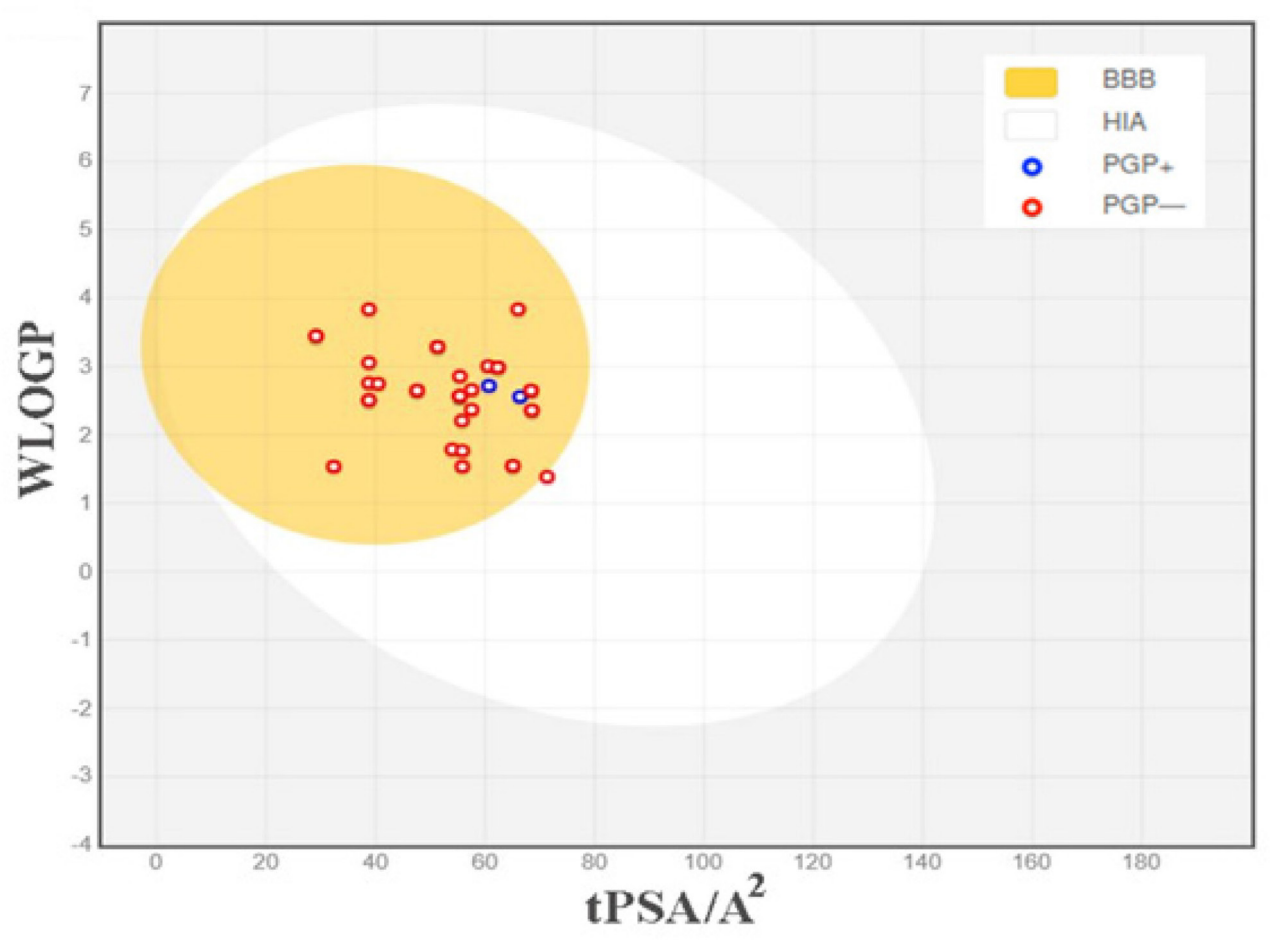
Predicted BOILED-Egg diagram of the selected compounds. BBB - Blood Brain Barrier, HIA - Human Intestinal Absorption, PGP^+^ - substrate of P-glycoprotein and PGP^-^ - non-substrate of P-glycoprotein

Literature mining and IMPPAT database searches identified 145 bioactive compounds. Of these, 82 bioactive compounds were identified in the fruit and 63 were identified in the roots of *P. longum* (Supplementary Table 1). Subsequently, all 145 compounds underwent the Swiss ADME analysis. To identify potential drug-like ligands, compounds were screened based on specific criteria, including oral bioavailability (OB) ≥ 30% and compliance with Lipinski's rule of 5. Swiss ADME assessment of *P. longum* compounds identified 33 potential drug-like ligands (Table 1), with 17 originating from the fruit and 16 from the root. The BOILED-Egg plot (Figure 1), incorporating the topological polar surface area (TPSA) and logarithm of the partition coefficient between n-octanol and water (Log PO/W), suggested that the tested compounds possessed favorable characteristics in terms of toxicity and drug-likeness. Molecular property analysis indicated that all 33 compounds fell within the appropriate range of Log P values, typically between -2 and 5. Furthermore, these compounds exhibit lower molecular weights, generally below 500-600 Da, indicating their potential for efficient permeability across the gastrointestinal tract (Human Intestinal absorption, HIA) and the blood-brain barrier (BBB). Among the phytocompounds, most are non-substrates for P-glycoprotein (PGP) and therefore can avoid P-gp efflux.

### Potential targets of* P. longum* and lung cancer

**Figure 2:**
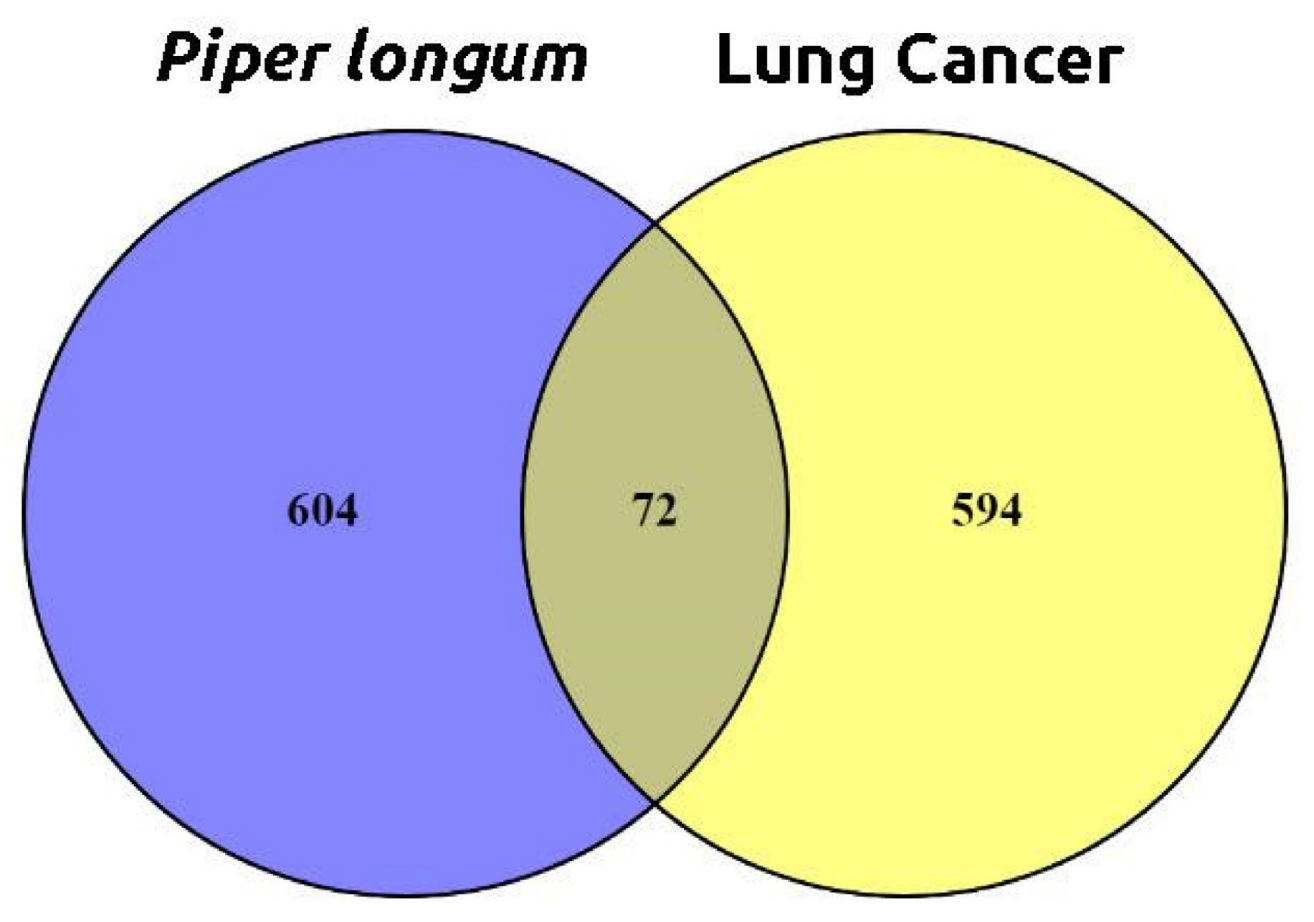
Intersecting targets between the active phytochemical potential protein targets in *P. longum* and Lung cancer-related genes.

After ADME screening, the bioactive compounds were subjected to target prediction using SwissTargetPrediction, SuperPred, and DIGEP-Pred databases, resulting in 676 target predictions. A comprehensive search using the keyword "Lung Cancer" in databases such as the Therapeutic Target Database, OMIM, and GeneCards resulted in a total of 666 target genes. When examining the target genes associated with *P. longum* and lung cancer, an overlap of 72 targets was identified and it is depicted in Figure 2.

### Protein-Protein interaction (PPI) network construction and analysis

**Figure 3:**
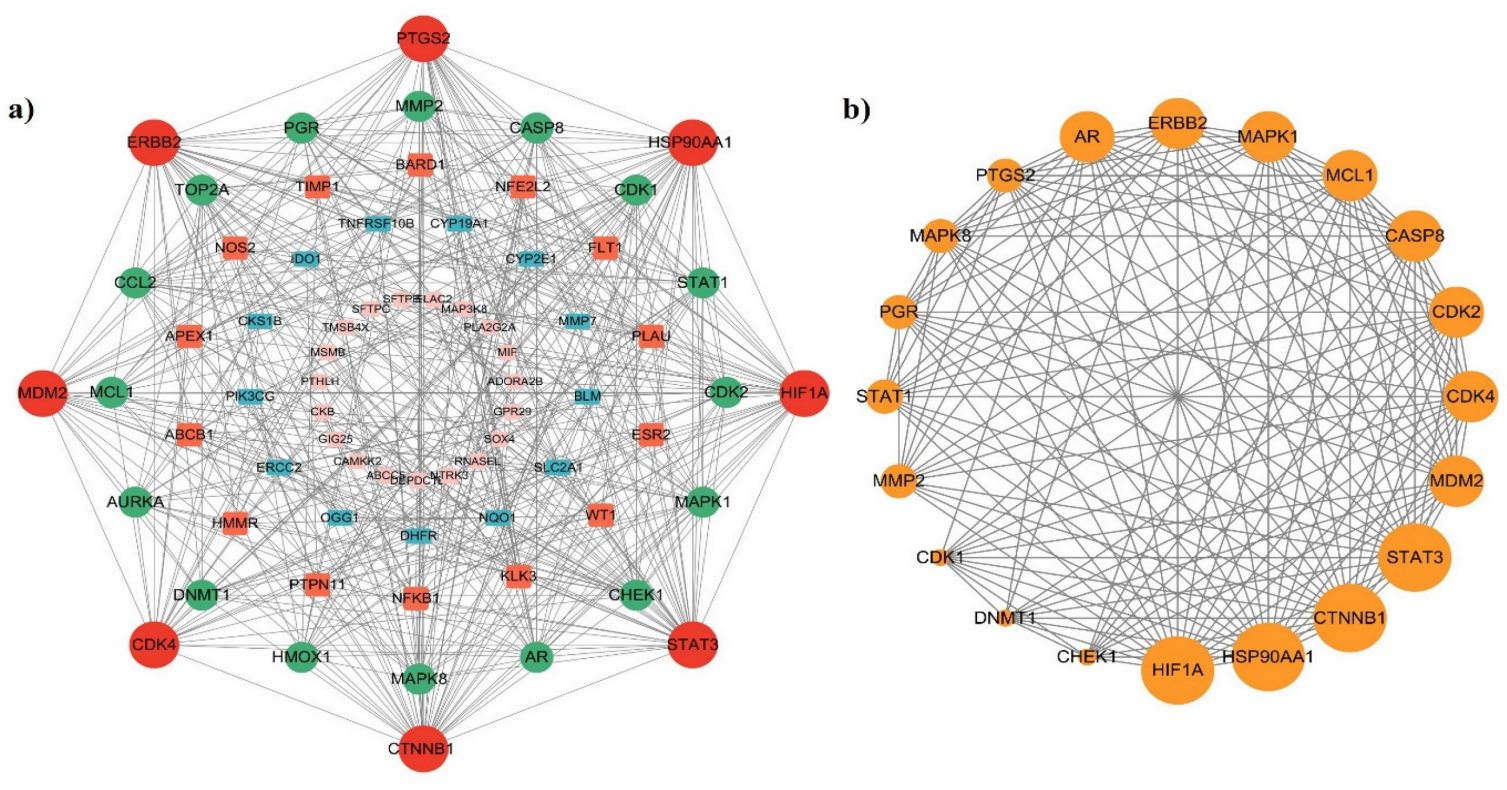
(a) The PPI network of the 72 intersecting targets and (b) The PPI network of the top 20 target genes were constructed.

The STRING database was used to create a protein-protein interaction (PPI) network using overlapping targets (Figure 3a). The resulting network comprises 72 nodes and 384 edges. To further analyze the network, the nodes were sorted based on their degrees, and a PPI network graph was created for the top 20 genes using the Cytoscape tool, as depicted in Figure 3b. The top 20 identified genes were CTNNB1, STAT3, HIF1A, HSP90AA1, ERBB2, PTGS2, MDM2, CDK4, CASP8, MAPK1, CDK2, MAPK8, AR, PGR, MMP2, STAT1, MCL1, CDK1, CHEK1, and DNMT1. The top 20 PPI network genes had 20 nodes, 155 edges, and an average number of neighbors of 15.50. The degree centrality (DC), betweenness centrality (BC), and closeness centrality (CC) values for the top 20 genes are shown in Supplementary Table 2. DC measures the number of direct connections that a node has. BC quantifies the number of times a node acts as a bridge along the shortest path between two other nodes. CC measures the closeness of a node to all other nodes in the network. It is the reciprocal of the average shortest path distance from node to all other nodes 30. Among the top 20 nodes, five (CTNNB1, STAT3, HIF1A, HSP90AA1, and ERBB2) exhibited DC, BC, and CC values that surpassed the average values. Therefore, these genes are potential lung cancer targets associated with *P. longum* infection. Table 2 lists the DC, BC, and CC values for the top five nodes.

### C-T-D Network Construction and Analysis

**Figure 4:**
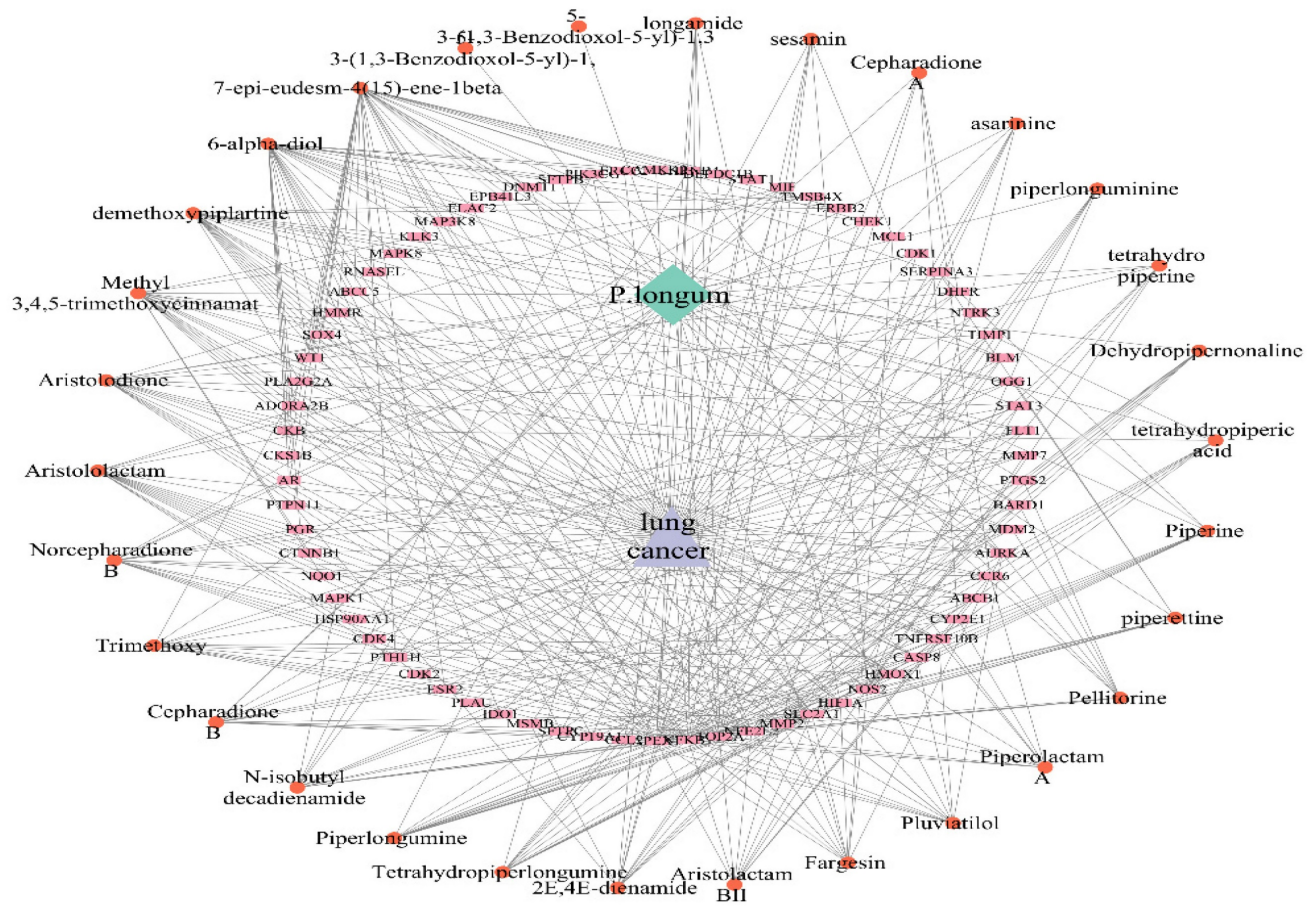
Compound - Target - Disease network of *P. longum* and lung cancer.

The Cytoscape tool was used to build a compound-target-disease network to examine the intricate relationships between compounds, targets, and diseases. The C-T-D network is illustrated in Figure 4 It contains 104 nodes and 423 edges with an average neighbor size of 8.135. Supplementary Table 3 shows the degree, betweenness, and closeness centralities of the top ten bioactive compounds linked to lung cancer. Among the bioactive compounds analyzed, 7-epi-eudesm-4(15)-ene-1beta, demethoxypiplartine, methyl 3,4,5-trimethoxycinnamate, 6-alpha-diol, and aristolodione showed the highest degrees, in the range of 15-32. Table 3 lists the DC, BC, and CC values of the top five bioactive substances with the highest node degrees.

### Gene Ontology (GO) enrichment and KEGG pathway Analysis

**Figure 5:**
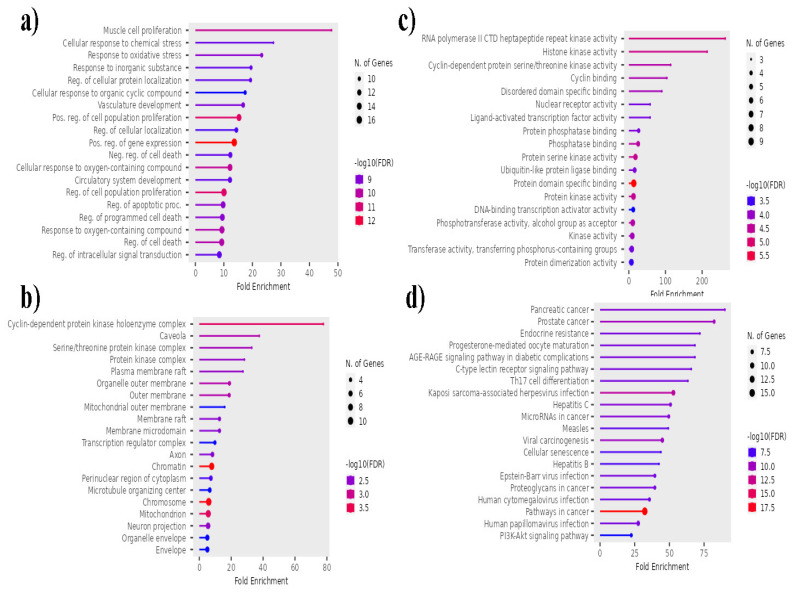
Top 20 GO terms and KEGG pathways associated with *P. longum* and lung cancer. a) Molecular Functions, b) Cellular Components c) Biological Processes and d) KEGG pathway.

*P. longum* targets were subjected to functional annotation and enrichment analysis, which revealed significant biological functions related to its phytochemical components. The targets linked to the phytochemical components found in the roots and fruits of *P. longum* were associated with a variety of biological processes (BP), including the regulation of cell death, muscle cell proliferation, cellular response to oxygen-containing compounds, and positive regulation of gene expression, according to Gene Ontology (GO) enrichment analysis. Targets for cellular components (CC) include mitochondria, cyclin-dependent protein kinase holoenzyme complex, chromatin, chromosomes, outer membrane, and other cellular components. Additionally, the molecular functions (MF) of the targets included binding to specific protein domains, RNA polymerase II CTD heptapeptide repeat kinase activity, histone kinase activity, protein kinase activity, protein serine kinase activity, and other activities. With an FDR cutoff of 0.05, 166 GO terms under MF, 143 under CC, and 1001 under BP were associated with the top 20 genes (Figure 5). The top 20 genes were connected to 158 pathways according to KEGG pathway analysis. Important pathways include those in cancer, Kaposi sarcoma-associated herpesvirus infection, prostate cancer, viral carcinogenesis, hepatitis C, pancreatic cancer, and microRNAs in cancer. Figure 5 displays the outcomes of the GO enrichment and KEGG pathway analyses.

### Expression of unique genes

**Figure 6:**
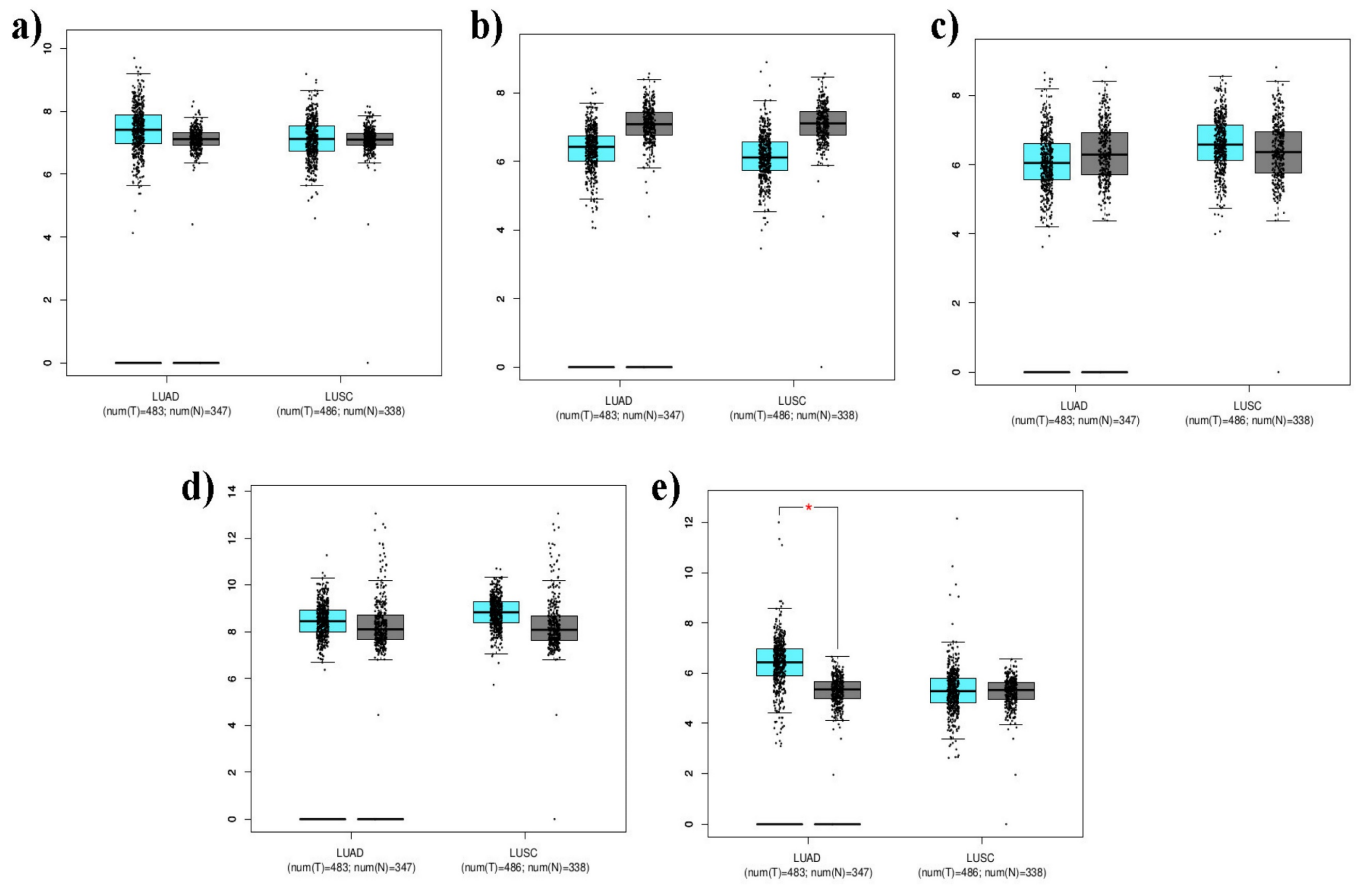
Box plots encode the 5-lung cancer associated gene expression levels [a)CTNNB1, b) ERBB2, c) HIF1A, d) HSP90AA1, e) STAT3] in LUAD and LUSC compared with normal cells. Tumor tissues are marked in cyan color and noncancerous or normal tissues are marked in grey color.

The expression levels of the top five lung cancer genes, CTNNB1, STAT3, HIF1A, HSP90AA1, and ERBB2, were determined by comparing them with the LUAD and LUSC datasets using the GEPIA2 web server (Figure 6). The TCGA_LUAD dataset contains expression data from 483 tumor tissues and 347 normal tissues associated with LUAD. The TCGA_LUSC dataset contained expression data from 486 tumor tissues and 338 normal tissues associated with LUSC. In the box plot, tumor tissues are marked in cyan, whereas normal tissues are marked in gray. The presence of a red asterisk indicates that a statistically significant p-value (less than 0.01) was calculated, suggesting a potentially meaningful difference in gene expression between tumor and normal tissues. The expression levels of the genes are represented on the y-axis using the mean log2 (TPM +1) values. This form of transformation is often used in gene expression analysis to stabilize variance across different expression levels. The results yielded that the above set lung associated cancer genes showed significant expressions with a p-value <0.01. ERBB2 was significantly upregulated and overexpressed in both TCGA_LUAD and _LUSC datasets. The expression levels of CTNNB1, STAT3, HIF1A, and HSP90AA1 were downregulated (Figure 6).

### Unique genes and their survival analysis

**Figure 7:**
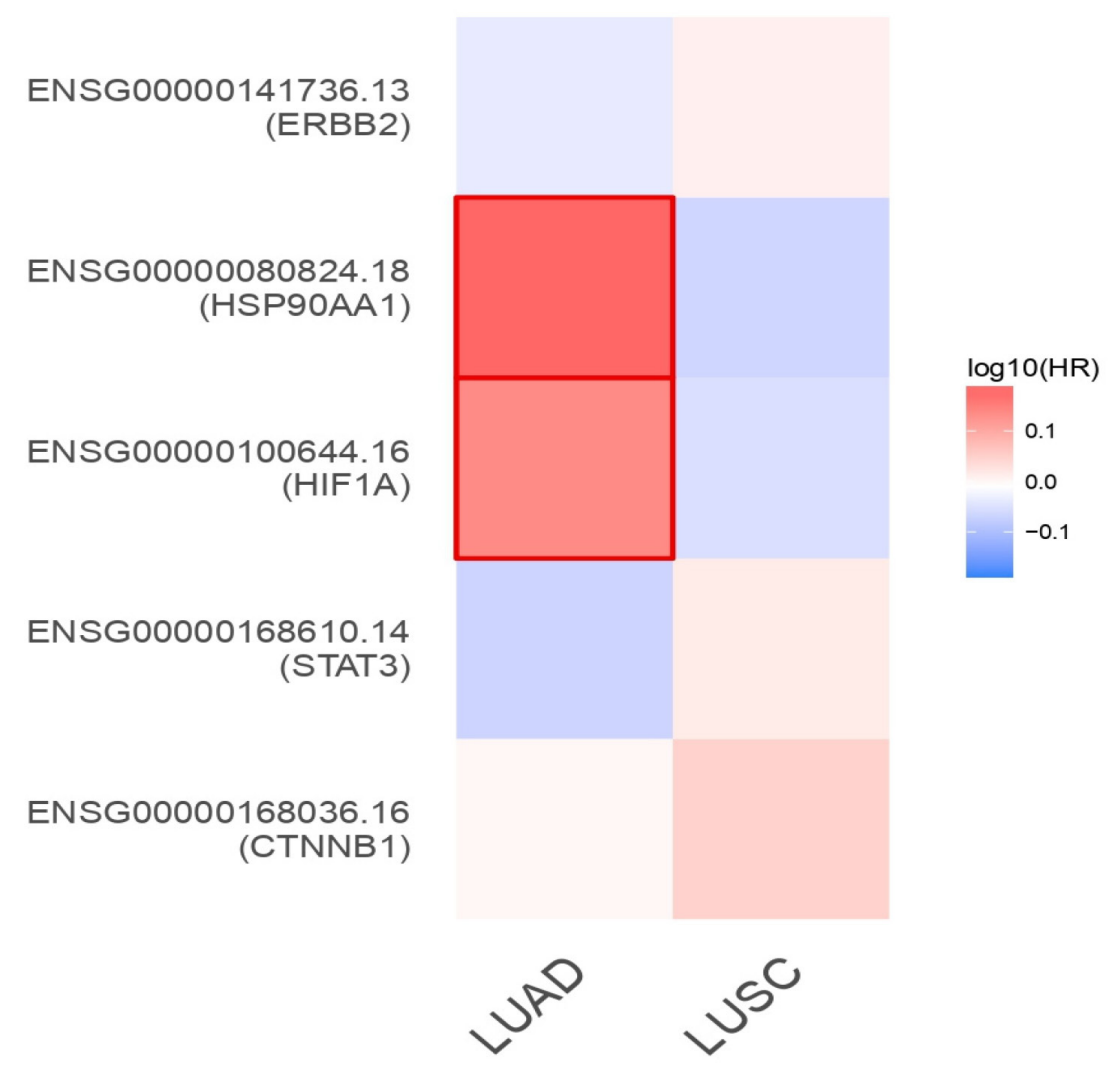
The survival heat map represents the prognostic impacts of unique gene expression levels based on the TCGA_LUAD and TCGA_LUSC datasets. The heat map represents the hazard ratios in log10 scale for the lung cancer associated genes. Red color represents higher risks, blue color indicates lower risks. The darkened rectangular frames indicate the significant favorable and unfavorable results in prognostic analyses.

Using GEPIA2, survival analysis was performed by comparing the gene expression levels determined by survival heat map analysis against TCGA_ LUAD and LUSC (Figure 7). Among the 5-lung cancer associated genes, HSP90AA1 and HIF1A were highly expressed in LUAD and LUSC datasets. However, all other genes, namely, CTNNB1, STAT3, and ERBB2, showed negligible expression in both the LUAD and LUSC datasets (Figure 7). A survival heat map is a visual representation of survival data that displays the relationship between certain variables (in this case, the top five lung cancer-associated genes) and survival outcomes (such as overall survival time) for different individuals or groups.

**Figure 8:**
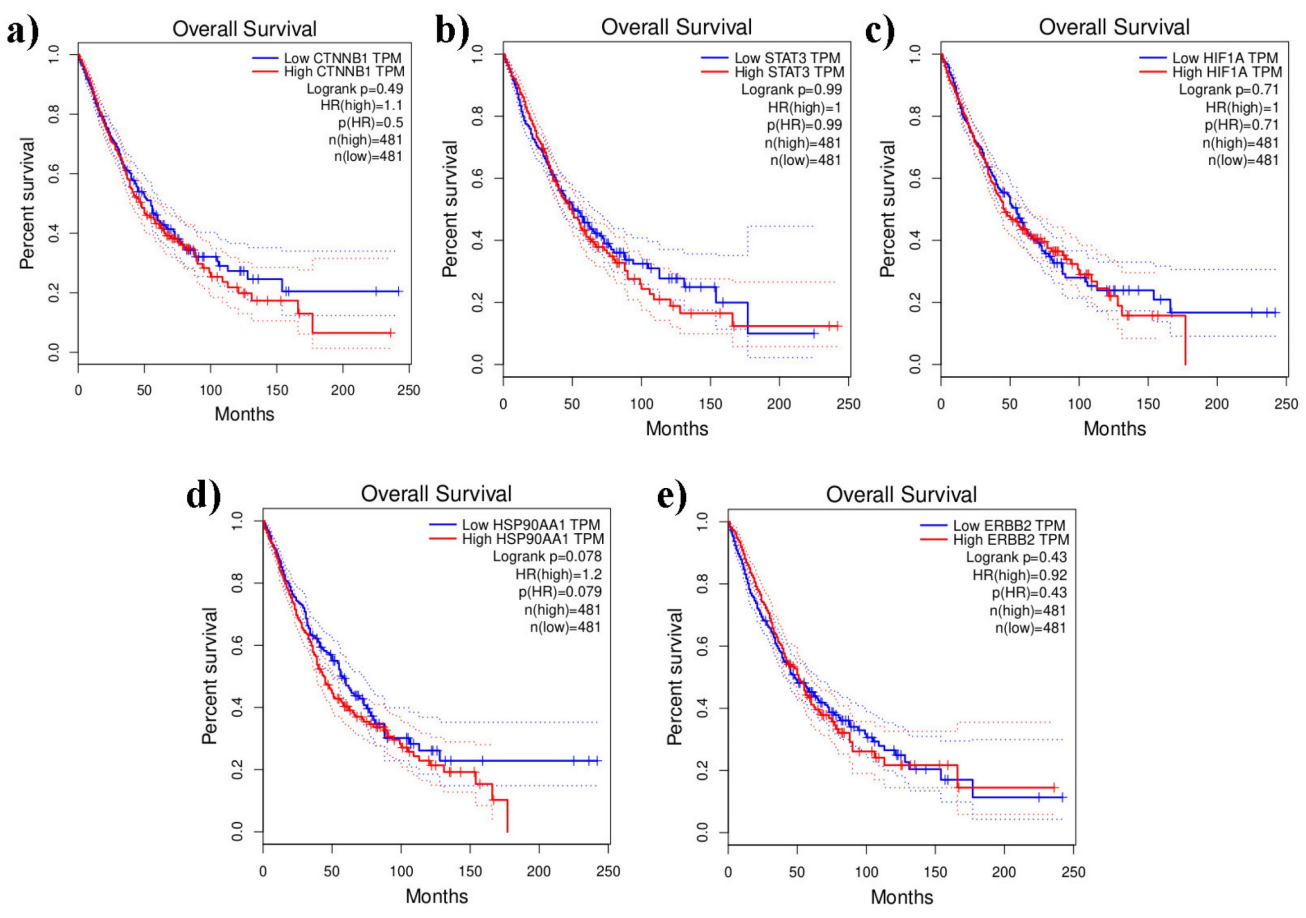
Lung cancer associated unique genes and their prognostic value is represented by overall survival analyses (Kaplan - Meier plotters and logrank tests) based on TCGA_LUAD and _LUSC visualized by GEPIA2. The dashed lines denote upper and lower confidence intervals. a) CTNNB1, b) ERBB2, c) HIF1A, d) HSP90AA1, e) STAT3.

Prognostic impact aids in understanding which genes are associated with better or worse survival outcomes and can help in identifying potential biomarkers for prognosis and treatment strategies. The Kaplan-Meier plotter shows that the distinct genes were substantially connected with lung cancer patients, with a p-value of 0.05, for the overall survival of lung cancer patients. In addition, certain genes with high expression and associations with survival in patients with LUAD and LUSC included CTNNB1(HR = 1.1; P = 0.5), STAT3 (HR = 1; P = 0.99), HIF1A (HR = 1; P = 0.71), and HSP90AA1 (HR = 1.2; P = 0.079). Additionally, the expression of the ERBB2 gene was associated with lower prognoses and lower survival rates in LUAD and LUSC patients (Figure 8). The heatmap results indicated that both of these genes (HSP90AA1 and HIF1A) at diagnosis can be considered undesirable prognostic genes and may lower the overall survival of LUAD- and LUSC-infected individuals (Figure 8).

### Molecular docking

**Figure 9:**
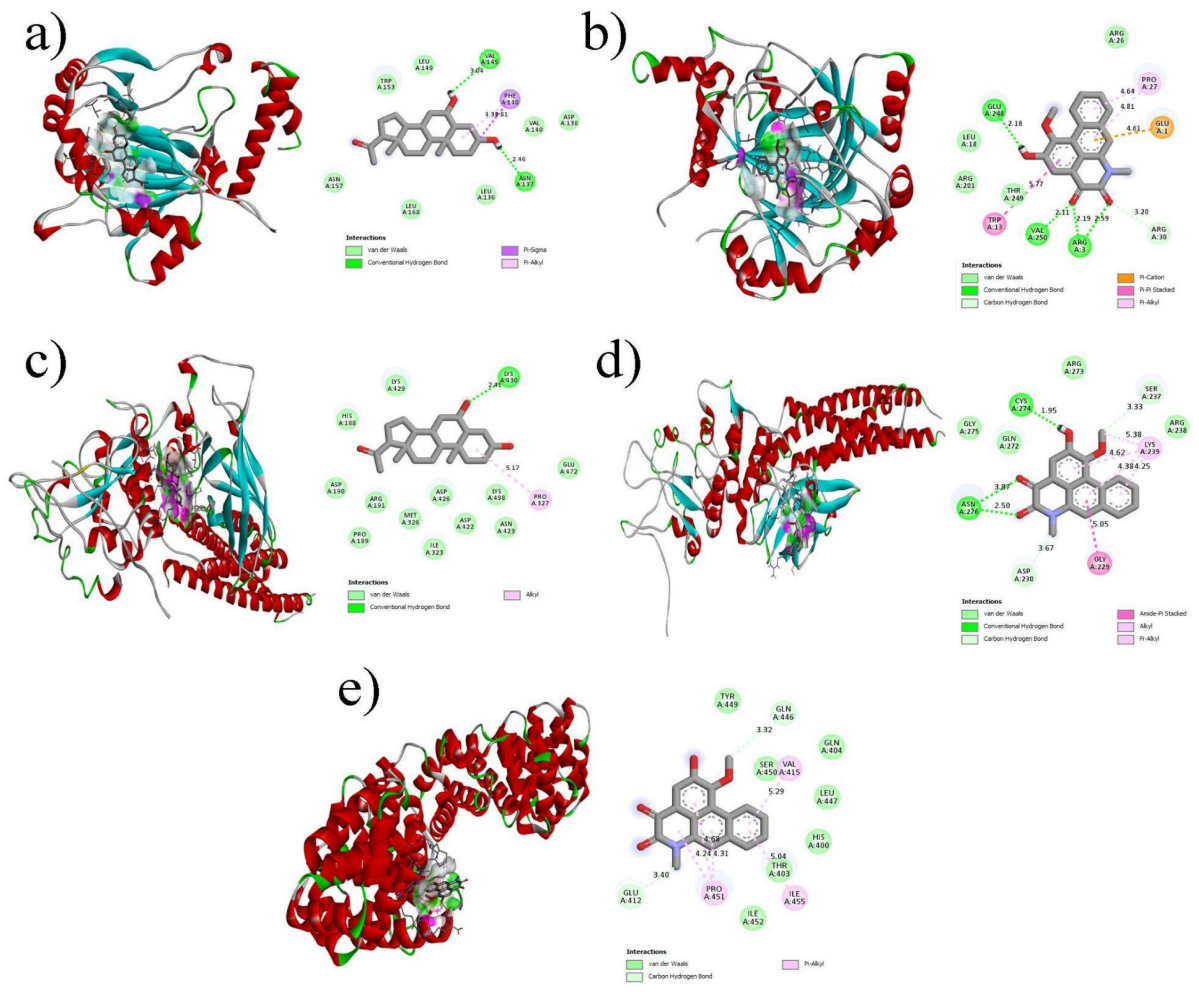
Molecular docking results of key Bioactive of *P.longum* with lung cancer core targets (a) CTNNB1 docked with Aristolodione (b) HIF1A docked with Aristolodione (c) STAT3 docked with 6-alpha-diol (d) STAT3 docked with Aristolodione (e) HIF1A docked with 6-alpha-diol.

The interactions between the components of the five hub genes and the phytochemicals were examined using molecular docking. Molecular docking studies involved the use of structures obtained from the Protein Data Bank (PDB), namely 1JDH 31, 1BG1 32, 3KCX 33, 3Q6N 34, and 1N8Z 35, which correspond to hub genes CTNNB1, STAT3, HIF1A, HSP90AA1, and ERBB2, respectively. Table 4 presents the top five ligand binding affinity values for each hub gene. Among the observed results, six protein-ligand complexes displayed binding affinities below -7 kcal/mol. kcal/mol), HSP90AA1_6-alpha-diol (-7.1 kcal/mol), STAT3_Aristolodione (-7.2 kcal/mol), CTNNB1_Aristolodione (-7.2 kcal/mol), STAT3_6-alpha-diol (-7.5 kcal/mol), HIF1A_Aristolodione (-7.8 kcal/mol), and HIF1A_6-alpha-diol complex (-7.9 kcal/mol). Figure 9 illustrates the 3D and 2D interaction plots of the protein-ligand complexes with the top five binding affinities.

In Figure 9a, the interactions between HIF1A and 6-alpha-diol are clearly depicted. The complex was stabilized by two hydrogen bonds (ASN137 and VAL145), one π-sigma bond (PHE148), one π-alkyl bond (PHE148), and several van der Waals interactions. In the case of the HIF1A_Aristolodione complex (Figure 9b), three hydrogen bonds formed by ARG3, VAL250, and GLU248, one π-π stacking interaction (TRP13), one π-cation (GLU1), and one π-alkyl bond (PRO27) were observed. Figure 9c shows the interactions between STAT3 and 6-alpha-diol, involving one hydrogen bond (LYS430), one alkyl bond (PRO327), and numerous van der Waals interactions. In contrast, the STAT3_Aristolodione complex (Figure 9d) exhibited three hydrogen bonds (one by CYS274 and two by ASN276), one amide-pi stacked interaction (GLY229), two alkyl bonds (LYS239), and two π-alkyl bonds (LYS239). Finally, the CTNNB1_Aristolodione complex (Figure 9e) was stable because of the formation of five π-alkyl bonds (one by VAL415, three by PRO451, and one by ILE455). The presence of multiple non-bonded interactions between the hub genes and the phytochemical constituents of *P. longum* suggests that these constituents may possess significant anticancer activity against lung cancer.

## Discussion

Network pharmacology offers a promising and scientifically rigorous approach to identify potential therapeutic interventions for lung cancer. This emerging field combines bioinformatics with experimental methodologies to construct comprehensive "compound-target/disease-gene" biomolecular networks. By analyzing these networks, network pharmacology enables the exploration of molecular interactions at different biological scales, providing insights into both the adverse and beneficial effects of drugs. Additionally, this approach revealed the underlying mechanisms of synergy among conventional medications, shedding light on their therapeutic properties for the treatment of diseases 36.

By analyzing the phytochemical constituents present in the fruit and roots of *P. longum*, this study successfully identified several noteworthy genes associated with cancer and potential anticancer substances. Specifically, the investigation revealed a connection between the phytochemical constituents and the genes CTNNB1, STAT3, HIF1A, HSP90AA1, and ERBB2, which have established links to lung cancer and play essential roles in the critical cellular pathways involved in disease progression. These findings strongly suggest that *P. longum* possesses properties that hold promise for alleviating lung cancer, thereby emphasizing its potential as a valuable therapeutic agent.

CTNNB1, also known as β-catenin-interacting protein 1, represses β-catenin transactivation and plays a significant role in the development of lung tumors 37. Signal Transducer and Activator of Transcription 3 (STAT3) revival has been observed in non-small cell lung cancer (NSCLC) patient samples. STAT3 plays a pivotal role in driving tumor-promoting inflammation and evasion of antitumor immunity 38. Hypoxia-inducible factor-1α (HIF-1α) is involved in tumor cell metastasis because it is a crucial transcription factor that regulates oxygen homeostasis 39. The AKT/glycogen synthase kinase 3 (GSK3) pathway, which functions downstream of sphingosine kinase-1 (SPHK-1), is involved in hypoxia-induced HIF-1 stability 40. HSP90AA1 (heat shock protein 90 alpha family class A member 1) plays a significant role in NSCLC regulation. Studies have also suggested that the HSP90AA1 protein product HSP90α plays a key role in regulating tumor invasion and migration 41. Erythroblastic oncogene B (ERBB2) has been identified as a marker for pancreatic malignancies, breast carcinomas, and gastric cancers. It is also known as human epidermal growth factor receptor 2 (HER2) 42. The HER2 gene encodes tyrosine kinase receptors, the modifications of which are known to cause carcinogenesis. HER2 modifications, including elaboration, deviation, and overexpression, have been noted in gastric and breast cancers 43.

GO enrichment analysis of the common genetic goals shared by lung cancer and the phytochemical constituents of *P. longum* revealed several GO biological pathways that were dysregulated in lung cancer. These pathways include how cells respond to the stress created by chemicals, the response to oxidative stress, regulation of intracellular signaling, and favorable control of cell population growth. Targeting these pathways holds potential for the development of therapeutics and is crucial for understanding the molecular mechanisms underlying lung cancer. Notably, oxidative stress, induced by factors such as smoking and air pollutants, contributes to the synthesis of pneumonia mediators in pulmonary epithelial cells that trigger carcinogenic mechanisms 44. Moreover, deactivation of the Wnt/β-catenin pathway, mediated by disheveled (Dsh) proteins, leads to accumulation of β-catenin in the cytosol. The gathered β-catenin is translocated into the nucleus, where it forms complexes with transcription factors, including T-cell factor family proteins (TCFs). These transcription factors are responsible for the activation of genes such as cyclin D1 and c-Myc, which are oncogenes involved in tumorigenesis and cell proliferation 45.

KEGG pathway analysis identified common genes with various KEGG pathways, including pathways such as cancer, microRNAs, proteoglycans, cancer, transforming growth factor-beta (TGF-beta) pathway, and PI3K-Akt pathway. An important relationship between these pathways provides valuable insights into the underlying molecular mechanisms involved in the development and progression of lung cancer. They play crucial roles in cell division, proliferation, apoptosis, and survival, and their dysregulation is well-documented in lung cancer. MicroRNAs (miRNAs), a family of small non-coding RNAs composed of 21-25 nucleotides, exert their regulatory effects by binding to complementary sites on target mRNAs. This communication is responsible for the inhibition of mRNA translation and promotion of mRNA degradation, thereby facilitating the post-transcriptional regulation of gene expression.

Glycosaminoglycans and proteoglycans are the major components of the Extracellular Matrix (ECM) that directly or indirectly interact with various cytokines, growth factors, adhesion molecules, and glycoproteins. This interaction contributes to cancer-related processes such as angiogenesis, proliferation, invasion, and metastasis 46. The TGF-β signaling pathway, which is responsible for epithelial-mesenchymal communication during lung branching and alveolarization, is implicated in pulmonary diseases 47. The PI3K-Akt signaling pathway involves a heterodimeric protein consisting of p110 catalytic and p85 regulatory subunits. AKT, a serine/threonine kinase, comprises a C-terminal tail domain and core kinase domain containing a threonine residue (T308) 48. NSCLC commonly activates the PI3K pathway, which is critical for oncogenesis as it promotes cell survival, growth, proliferation, and migration 49.

Molecular docking is a computational method used in network pharmacology and drug discovery to forecast and examine atomic-level interactions between small molecules (ligands) and target proteins (receptors). They play a crucial role in elucidating the potential therapeutic effects of compounds and their mechanisms of action by determining the preferred orientation of a ligand to a target to form a stable complex. In the context of lung cancer research, molecular docking experiments were conducted using the top five compounds derived from *P. longum* and the top five lung cancer-related genes. The results revealed that 6-alpha-diol exhibited the highest affinity for HIF1A, suggesting that HIF1A may be a suitable target for lung cancer treatment. Furthermore, the strong binding of 6-alpha-diol to HIF1A suggests that it may possess pharmacological activity against HIF1A-mediated pathways. In particular, 6-alpha-diol has the potential to modulate HIF1A signaling in hypoxic environments relevant to lung cancer, thereby influencing processes, such as angiogenesis, metabolism, and cell survival.

Network pharmacology and molecular docking approaches have been developed in recent years and are widely employed to investigate the key targets and underlying mechanisms responsible for the anticancer properties of various medicinal plants. Iksen *et al.* 46 used a network pharmacology approach to identify lung cancer targets associated with aspileterin-derived steroidal saponins. Their study revealed significant anticancer targets including IL2, FGF2, HSP90AA1, VEGFA, and STAT3. Furthermore, molecular docking studies demonstrated the strong binding affinity of aspileterin A for STAT3. Another study conducted by Cheng *et al.* 49 examined the anticancer effects of Qishan formula against lung adenocarcinoma. This study identified several major anticancer targets, including AKT1, HRAS, PIK3CA, HSP90AA1, MAKP1, STAT3, MAPK3, PIK3R1, TP53, and SRC. Among these targets, HSP90AA1 exhibited high-affinity binding (up to 10 kcal/mol) to six compounds present in the Qishan formula. Additionally, Zhou *et al.* 50 conducted a study on *Camellia nitidissima* C.W.Chi and identified five phytochemical constituents (3'4-O-dimethylcedrusin, eriodictyol, quercetin, kaempferol, and luteolin) that showed high-affinity binding to four lung cancer targets (CCND1, AKT1, SRC, and EGFR). The findings of this study align closely with those of the aforementioned studies, particularly with regard to the identified anticancer targets.

Overall, the network pharmacology analysis of *P. longum* fruits and roots provides valuable insights into potential therapeutic targets for lung cancer. However, further research is necessary to validate the findings of our analysis and establish the safety and efficacy of P. longum-based therapeutics. Rigorous pre-symptomatic and clinical studies should be conducted to ensure the safety and effectiveness of potential treatments in human subjects after their identification.

## Conclusions

In this study, a network pharmacology approach was employed to identify potential bioactive substances and their corresponding targets for the treatment of lung cancer. A total of 145 bioactive compounds were identified by a literature review and the IMPPAT database, of which 33 showed potential anticancer properties. These compounds were screened using Swiss ADME considering their drug-like properties and adherence to specific restrictions. Subsequent filtering with Swiss Target, SuperPred, and DIGEP-Pred resulted in 37 compounds with 676 targets, of which 72 overlapped with 666 lung cancer gene targets. To assess the key compound targets and establish the main hub nodes for the *P. longum* lung cancer-curing effect, we constructed a protein-protein interaction network using STRING and Cytoscape software. Key parameters include the degree, betweenness centrality, and closeness centrality. Notably, substances such as 7-epi-eudesm-4(15)-ene-1beta, demethoxypiplartine, methyl 3,4,5-trimethoxycinnamate, 6-alpha-diol, and aristolodione exhibited higher degrees of inhibition, indicating their potential therapeutic value in lung cancer. Additionally, this study identified dysregulated GO biological pathways associated with lung cancer, including how cells respond to the stress created by chemicals, the response to oxidative stress, regulation of intracellular signaling, and favorable control of cell population growth. These pathways play vital roles in understanding the molecular mechanisms underlying lung cancer and are potential targets for therapeutic development. KEGG pathway enrichment analysis revealed the transforming growth factor-beta pathway, PI3K-Akt pathway, miRNAs, proteoglycans, and cancer, shedding further light on the molecular mechanisms associated with lung cancer development and progression. Furthermore, docking analysis demonstrated the potential of 6-alpha-diol and similar chemicals from *P. longum* in targeting HIF1A. However, presymptomatic and clinical studies are essential to confirm the safety and efficacy of these potential therapeutic agents in humans. Nevertheless, our study demonstrated the promising application of network pharmacology in the discovery of potential anti-lung cancer compounds.

## Supplementary Material

Supplementary tables.

## Figures and Tables

**Table 1:** ADME properties of the selected phytochemicals of *P. longum*

Name of the compound	MW	MR	Rotatable bonds	XLOGP3	H-bond acceptors	H-bond donors	Bioavailability Score
Norcepharadione B	307.3	90.02	2	3.16	4	1	0.55
Piperine	285.34	85.47	4	3.46	3	0	0.55
Aristolodione	307.3	90.45	1	3.02	4	1	0.55
Aristolactam BII	279.29	83.75	2	3.28	3	1	0.55
Dehydropipernonaline	339.43	104.23	7	4.7	3	0	0.55
Piperlongumine	317.34	89.47	6	2.07	5	0	0.55
Pluviatilol	356.37	92.45	3	2.48	6	1	0.55
Aristololactam	293.27	83.32	1	3.12	4	1	0.55
Fargesin	370.4	96.92	4	2.81	6	0	0.55
Pellitorine	223.35	71.47	9	4.39	1	1	0.55
Aristolactam AII	265.26	79.28	1	2.95	3	2	0.55
longamide	351.98	67.28	2	1.23	3	2	0.85
tetrahydropiperic acid	222.24	58.47	5	2.54	4	1	0.85
piperettine	311.37	94.61	5	4.11	3	0	0.55
asarinine	354.35	90	2	2.68	6	0	0.55
piperlonguminine	273.33	78.77	6	4.32	3	1	0.55
demethoxypiplartine	287.31	82.98	5	2.1	4	0	0.55
N-isobutyl decadienamide	223.35	71.47	9	3.69	1	1	0.55
tetrahydro piperine	289.37	85.63	6	3.52	3	0	0.55
sesamin	354.35	90	2	2.68	6	0	0.55
Cepharadione B	321.33	94.92	2	3.34	4	0	0.55
Norcepharadione B	307.3	90.02	2	3.16	4	1	0.55
Piperine	285.34	85.47	4	3.46	3	0	0.55
Piperlonguminine	273.33	78.77	6	4.32	3	1	0.55
Methyl 3,4,5-trimethoxycinnamate	252.26	66.91	6	2.07	5	0	0.55
Cepharadione A	305.28	88	0	3.21	4	0	0.55
Piperolactam A	265.26	79.28	1	2.95	3	2	0.55
5-[3-(1,3-Benzodioxol-5-yl)-1,3,3a,4,6,6a-hexahydrofuro[3,4-c]furan-6-yl]-1,3-benzodioxole	354.35	90	2	2.68	6	0	0.55
Tetrahydropiperlongumine	218.29	69.49	4	2.06	2	1	0.55
Trimethoxy cinnamoyl-piperidine	289.33	83.46	5	1.95	4	0	0.55
6-alpha-diol	308.46	88.25	0	3.17	3	3	0.55
2E,4E-dienamide	343.17	84.03	5	4.1	3	1	0.55
7-epi-eudesm-4(15)-ene-1beta	238.37	71.58	1	2.63	2	2	0.55

**Table 2:** The DC, BC and CC values of the hub genes.

Gene	Degree	Betweenness	Closeness
CTNNB1	42.0	536.3216	0.70408165
STAT3	39.0	415.04984	0.6831683
HIF1A	37.0	601.5508	0.6699029
HSP90AA1	35.0	279.21713	0.6448598
ERBB2	33.0	225.435	0.6330275

**Table 3:** The DC, BC and CC values of the compounds with high node degrees.

Compound	Degree	Betweenness	Closeness
7-epi-eudesm-4(15)-ene-1beta	33.0	820.94586	0.48356807
demethoxypiplartine	20.0	242.6487	0.43096235
Methyl 3,4,5-trimethoxycinnamate	19.0	244.99773	0.4273859
6-alpha-diol	31.0	720.89215	0.47465438
Aristolodione	17.0	164.20059	0.42040816

**Table 4:** Top 5 binding affinity values of ligands with the hub genes.

Target gene and Ligand	Binding affinity (kcal/mol)
CTNNB1_6-alpha-diol	-6.5
CTNNB1_7-epi-eudesm-4(15)-ene-1beta	-6
CTNNB1_Aristolodione	-7.2
CTNNB1_Demethoxypiplartine	-6.4
CTNNB1_Methyl_3,4,5-trimethoxycinnamate	-5
STAT3_6-alpha-diol	-7.5
STAT3_7-epi-eudesm-4(15)-ene-1beta	-6.4
STAT3_Aristolodione	-7.2
STAT3_Demethoxypiplartine	-7
STAT3_Methyl_3,4,5-trimethoxycinnamate	-5.9
HIF1A_6-alpha-diol	-7.9
HIF1A_7-epi-eudesm-4(15)-ene-1beta	-6.4
HIF1A_Aristolodione	-7.8
HIF1A_Demethoxypiplartine	-6.7
HIF1A_Methyl_3,4,5-trimethoxycinnamate	-5.7
HSP90AA1_6-alpha-diol	-7.1
HSP90AA1_7-epi-eudesm-4(15)-ene-1beta	-6
HSP90AA1_Aristolodione	-6.9
HSP90AA1_Demethoxypiplartine	-6.6
HSP90AA1_Methyl_3,4,5-trimethoxycinnamate	-5.6
ERBB2_6-alpha-diol	-6.7
ERBB2_7-epi-eudesm-4(15)-ene-1beta	-5.5
ERBB2_Aristolodione	-7
ERBB2_Demethoxypiplartine	-6
ERBB2_Methyl_3,4,5-trimethoxycinnamate	-5

## Abbreviations

TTD: Therapeutic Target Database, PPI: protein-protein interaction
OMIM: Online Mendelian Inheritance in Man
IMPPAT: Indian Medicinal Plants, Phytochemistry and Therapeutics database
GO: Gene ontology
PDB: Protein data bank
TPSA: topological polar surface area
C-T-D: Compound to target
GSK3: AKT/glycogen synthase kinase 3
STAT3: Signal Transducer and Activator of Transcription 3
NSCLC: Non-small cell lung cancer
HIF-1α: Hypoxia-inducible factor-1α
SPHK-1: sphingosine kinase-1
ERBB2: Erythroblastic oncogene B
HER2: human epidermal growth factor receptor 2
TCFs: T-cell factor family proteins
TGF-beta: Transforming growth factor-beta
