# Supplementary material for: Deciphering the Anticancer Arsenal of Piper longum: Network Pharmacology and Molecular Docking Unveil Phytochemical Targets Against Lung Cancer
Source: Int J Med Sci. 2024 Jul 22;21(10):1915–28. doi: 10.7150/ijms.98393 (PMC11302554; doi:10.7150/ijms.98393)
Supplement: Supplementary file 1 — Supplementary tables. [file ijmsv21p1915s1.pdf]

**Supplementary Information**

Table S1: The list of compounds identified from the root and fruit of *P. longum*

Table S2: The DC, BC and CC values of the top 20 genes with high node degrees

Table S3: The DC, BC and CC values of the top 10 compounds with high node degrees

**Supplementary Information**

**Table S1: The list of compounds identified from the root and fruit of *P. longum***

| Compounds in fruit | Compounds in root |
|---|---|
| Hexadecane | 2-Tridecanone |
| Piperundecalidine | 1-Pentadecene |
| 2-Tridecanone | Cepharadione B |
| Octadecane | Norcepharadione B |
| 1-Pentadecene | Myrcene |
| Norcepharadione B | Piperine |
| Myrcene | Piperlonguminine |
| Piperine | Aristolodione |
| Aristolodione | Aristolactam BII |
| Aristolactam BII | Piperlongumine |
| p-Cymene | Decanal |
| 4'-Methoxyacetophenone | 1-Methylhexyl acetate |
| Dehydropipernonaline | Methyl 3,4,5-trimethoxycinnamate |
| Guineensine | Dodecanal |

| | |
|---|---|
| 2-Phenylethanol | Cepharadione A |
| Piperlongumine | beta-Cubebene |
| Pluviatilol | Cadinane |
| Eicosane | Heptadecane |
| Decanal | Pentadecane |
| 1-Methylhexyl acetate | Triacontane |
| Dodecanal | Tridecane |
| beta-Cubebene | 2-Nonanone |
| Cadinane | Eucalyptol |
| Pipernonaline | beta-Elemene |
| Aristololactam | alpha-Terpinyl acetate |
| Heptadecane | 2-Undecanone |
| Nonadecane | beta-Eudesmol |
| Heneicosane | Terpinolene |
| Pentadecane | (+)-alpha-Gurjunene |
| Tridecane | alpha-Muurolene |
| 2-Nonanone | alpha-Eudesmol |
| Eucalyptol | Humulene |
| beta-Elemene | gamma-Muurolene |
| Isobutyramide | (Z)-gamma-bisabolene |
| alpha-Terpinyl acetate | Elemol |
| 2-Undecanone | (+)-beta-Phellandrene |
| beta-Eudesmol | Linalool |
| (1R)-2-methyl-5-propan-2-ylbicyclo[3.1.0]hex-2-ene | alpha-Pinene |
| Fargesin | beta-Pinene |

| | |
|---|---|
| Pellitorine | alpha-Terpineol |
| Terpinolene | beta-Patchoulene |
| (+)-alpha-Gurjunene | delta-Cadinol |
| alpha-Muurolene | (-)-alpha-Cadinol |
| alpha-Eudesmol | 9-Eicosyne |
| Zingiberene | Caryophyllene oxide |
| Humulene | Piperolactam A |
| gamma-Muurolene | (Z)-beta-Ocimene |
| (Z)-gamma-bisabolene | gamma-Elemene |
| Elemol | delta-Elemene |
| (+)-beta-Phellandrene | alpha-Phellandrene |
| Linalool | beta-Caryophyllene |
| alpha-Pinene | (E)-beta-ocimene |
| Dihydrocarveol | Bornyl acetate |
| beta-Pinene | Camphene |
| alpha-Terpineol | Copaene |
| beta-Patchoulene | 5-[3-(1,3-Benzodioxol-5-yl)-1,3,3a,4,6,6a-hexahydrofuro[3,4-c]furan-6-yl]-1,3-benzodioxole |
| Aristolactam AII | Limonene |
| delta-Cadinol | Nerolidol |
| (-)-alpha-Cadinol | Tetrahydropiperlongumine |
| 9-Eicosyne | Trimethoxy cinnamoyl-piperidine |
| Caryophyllene oxide | 6-alpha-diol |
| (Z)-beta-Ocimene | 2E,4E-dienamide |
| gamma-Elemene | 7-epi-eudesm-4(15)-ene-1beta |

| | |
|---|---|
| delta-Elemene | |
| alpha-Phellandrene | |
| beta-Caryophyllene | |
| (E)-beta-ocimene | |
| Bornyl acetate | |
| Camphene | |
| Copaene | |
| 5-[3-(1,3-Benzodioxol-5-yl)-1,3,3a,4,6,6a-hexahydrofuro[3,4-c]furan-6-yl]-1,3-benzodioxole | |
| Limonene | |
| Nerolidol | |
| longamide | |
| tetrahydropiperic acid | |
| piperettine | |
| asarinine | |
| piperlonguminine | |
| demethoxypiplartine | |
| N-isobutyl decadienamide | |
| tetrahydro piperine | |
| sesamin | |

**Table S2: The DC, BC and CC values of the top 20 genes with high node degrees**

| S.NO | GENE | DEGREE | BETWEENNESS | CLOSENESS |
|---|---|---|---|---|

| | | | | |
|---|---|---|---|---|
| 1 | CTNNB1 | Degree: 42.0 | Betweenness: 536.3216 | Closeness: 0.70408165 |
| 2 | STAT3 | Degree: 39.0 | Betweenness: 415.04984 | Closeness: 0.6831683 |
| 3 | HIF1A | Degree: 37.0 | Betweenness: 601.5508 | Closeness: 0.6699029 |
| 4 | HSP90AA1 | Degree: 35.0 | Betweenness: 279.21713 | Closeness: 0.6448598 |
| 5 | ERBB2 | Degree: 34.0 | Betweenness: 310.8129 | Closeness: 0.6448598 |
| 6 | PTGS2 | Degree: 33.0 | Betweenness: 225.435 | Closeness: 0.6330275 |
| 7 | MDM2 | Degree: 27.0 | Betweenness: 92.45294 | Closeness:0.58474576 |
| 8 | CDK4 | Degree: 26.0 | Betweenness: 109.61591 | Closeness: 0.57983196 |
| 9 | CASP8 | Degree: 22.0 | Betweenness: 37.619987 | Closeness:0.57024795 |
| 10 | MAPK1 | Degree: 23.0 | Betweenness: 101.96046 | Closeness: 0.575 |
| 11 | CDK2 | Degree: 23.0 | Betweenness: 80.629715 | Closeness: 0.56557375 |
| 12 | MAPK8 | Degree: 23.0 | Betweenness: 55.634125 | Closeness: 0.5609756 |
| 13 | AR | Degree: 23.0 | Betweenness: 309.2111 | Closeness: 0.57024795 |

| | | | | |
|---|---|---|---|---|
| 14 | PGR | Degree: 21.0 | Betweenness: 55.176437 | Closeness: 0.56557375 |
| 15 | MMP2 | Degree: 21.0 | Betweenness: 48.341125 | Closeness: 0.5564516 |
| 16 | STAT1 | Degree: 22.0 | Betweenness: 118.963036 | Closeness: 0.54761904 |
| 17 | MCL1 | Degree: 18.0 | Betweenness: 16.44801 | Closeness: 0.53488374 |
| 18 | CDK1 | Degree: 22.0 | Betweenness: 137.07443 | Closeness: 0.5564516 |
| 19 | CHEK1 | Degree: 23.0 | Betweenness: 161.48833 | Closeness: 0.54330707 |
| 20 | DNMT1 | Degree: 16.0 | Betweenness: 13.377821 | Closeness: 0.5307692 |

**Table S3: The DC, BC and CC values of the top 10 compounds with high node degrees**

| S.NO | Compound Name | Degree | Betweenness | Closeness |
|---|---|---|---|---|
| 1 | 7-epi-eudesm-4(15)-ene-1beta | Degree: 33.0 | Betweenness: 820.94586 | Closeness: 0.48356807 |
| 2 | demethoxypiplartine | Degree: 20.0 | Betweenness: 242.6487 | Closeness: 0.43096235 |

| | | | | |
|---|---|---|---|---|
| 3 | Methyl 3,4,5-trimethoxycinnamate | Degree: 19.0 | Betweenness: 244.99773 | Closeness: 0.4273859 |
| 4 | 6-alpha-diol | Degree: 31.0 | Betweenness: 720.89215 | Closeness: 0.47465438 |
| 5 | Aristolodione | Degree: 17.0 | Betweenness: 164.20059 | Closeness: 0.42040816 |
| 6 | Aristolactam | Degree: 16.0 | Betweenness: 208.32811 | Closeness: 0.41700405 |
| 7 | Norcepharadione B | Degree: 15.0 | Betweenness: 125.49597 | Closeness: 0.41365463 |
| 8 | Piperlongumine | Degree: 13.0 | Betweenness: 69.643684 | Closeness: 0.40711462 |
| 9 | Cepharadione B | Degree: 13.0 | Betweenness: 87.37466 | Closeness: 0.40711462 |
| 10 | Fargesin | Degree: 11.0 | Betweenness: 55.324604 | Closeness: 0.4007782 |
